# Vitamin A Metabolism by Dendritic Cells Triggers an Antimicrobial Response against Mycobacterium tuberculosis

**DOI:** 10.1128/mSphere.00327-19

**Published:** 2019-06-05

**Authors:** Elliot W. Kim, Avelino De Leon, Zhichun Jiang, Roxana A. Radu, Adrian R. Martineau, Edward D. Chan, Xiyuan Bai, Wen-Lin Su, Dennis J. Montoya, Robert L. Modlin, Philip T. Liu

**Affiliations:** a Division of Dermatology, David Geffen School of Medicine, University of California, Los Angeles, Los Angeles, California, USA; b Department of Molecular Biology Institute, University of California, Los Angeles, Los Angeles, California, USA; c Barts and The London School of Medicine and Dentistry, Queen Mary University of London, London, United Kingdom; d Department of Medicine and Academic Affairs, National Jewish Health, Denver, Colorado, USA; e Division of Pulmonary and Critical Care Medicine, Taipei Tzu Chi Hospital, Buddhist Tzu Chi Medical Foundation, New Taipei, Taiwan; f UCLA and Orthopaedic Hospital Department of Orthopaedic Surgery, The Orthopaedic Hospital Research Center, Los Angeles, Los Angeles, California, USA; g Department of Molecular, Cell, and Developmental Biology, University of California, Los Angeles, Los Angeles, California, USA; h Stein Eye Institute and Department of Ophthalmology, David Geffen School of Medicine, University of California, Los Angeles, Los Angeles, California, USA; Washington University School of Medicine in St. Louis

**Keywords:** *Mycobacterium tuberculosis*, dendritic cells, transcellular metabolism

## Abstract

Epidemiological evidence correlates low serum vitamin A (retinol) levels with increased susceptibility to active tuberculosis (TB); however, retinol is biologically inactive and must be converted into its bioactive form, all-*trans* retinoic acid (ATRA). Given that ATRA triggers a Niemann-Pick type C2 (NPC2)-dependent antimicrobial response against Mycobacterium tuberculosis, we investigated the mechanism by which the immune system converts retinol into ATRA at the site of infection. We demonstrate that granulocyte-macrophage colony-stimulating factor (GM-CSF)-derived dendritic cells (DCs), but not macrophages, express enzymes in the vitamin A metabolic pathway, including aldehyde dehydrogenase 1 family, member a2 (ALDH1A2) and short-chain dehydrogenase/reductase family, member 9 (DHRS9), enzymes capable of the two-step conversion of retinol into ATRA, which is subsequently released from the cell. Additionally, mRNA and protein expression levels of ALDH1A2 and DC marker CD1B were lower in tuberculosis lung tissues than in normal lung. The conditioned medium from DCs cultured with retinol stimulated antimicrobial activity from M. tuberculosis-infected macrophages, as well as the expression of NPC2 in monocytes, which was blocked by specific inhibitors, including retinoic acid receptor inhibitor (RARi) or *N*,*N*-diethylaminobenzaldehyde (DEAB), an ALDH1A2 inhibitor. These results indicate that metabolism of vitamin A by DCs transactivates macrophage antimicrobial responses.

**IMPORTANCE** Tuberculosis (TB) is the leading cause of death by a single infectious agent worldwide. One factor that contributes to the success of the microbe is the deficiency in immunomodulatory nutrients, such as vitamin A (retinol), which are prevalent in areas where TB is endemic. Clinical trials show that restoration of systemic retinol levels in active TB patients is ineffective in mitigating the disease; however, laboratory studies demonstrate that activation of the vitamin A pathway in Mycobacterium tuberculosis-infected macrophages triggers an antimicrobial response. Therefore, the goal of this study was to determine the link between host retinol levels and retinoic acid-mediated antimicrobial responses against M. tuberculosis. By combining established *in vitro* models with *in situ* studies of lung tissue from TB patients, this study demonstrates that the innate immune system utilizes transcellular metabolism leading to activation between dendritic cells and macrophages as a means to combat the pathogen.

## Abstract (precis)

Tuberculosis (TB) is the leading cause of death by a single infectious agent worldwide. One factor that contributes to the success of the microbe is the deficiency in immunomodulatory nutrients, such as vitamin A (retinol), which are prevalent in areas where TB is endemic. Clinical trials show that restoration of systemic retinol levels in active TB patients is ineffective in mitigating the disease; however, laboratory studies demonstrate that activation of the vitamin A pathway in Mycobacterium tuberculosis-infected macrophages triggers an antimicrobial response. Therefore, the goal of this study was to determine the link between host retinol levels and retinoic acid-mediated antimicrobial responses against M. tuberculosis. By combining established *in vitro* models with *in situ* studies of lung tissue from TB patients, this study demonstrates that the innate immune system utilizes transcellular metabolism leading to activation between dendritic cells and macrophages as a means to combat the pathogen.

## INTRODUCTION

Globally, there are 1.7 billion people infected with Mycobacterium tuberculosis, the etiological agent of tuberculosis (TB), of which, approximately 10% will develop the active disease and the rest will remain latently infected (1). The incidence of TB is prominent in low-income areas where people can also suffer from malnutrition, resulting in lower systemic levels of immunomodulatory vitamins. Epidemiological studies have correlated low serum levels of retinol, the circulating form of vitamin A, with a 10-fold increased risk and susceptibility to TB (2–4). In addition, *in vitro* studies have demonstrated that stimulation of M. tuberculosis-infected macrophages with all-*trans* retinoic acid (ATRA), the bioactive hormonal form of vitamin A, induced antimicrobial activity against the pathogen (5–8). Collectively, these studies indicate an important role for the vitamin A system in the immune response against M. tuberculosis infection. However, for systemic retinol to influence immune responses at the site of infection, it must first be metabolized into ATRA.

We and others previously showed that treatment of M. tuberculosis-infected macrophages with ATRA results in antimicrobial activity (5, 7, 8). Our study demonstrated that at least one of the mechanisms driving the ATRA-triggered antimicrobial activity is the expression of the lipid transporter protein Niemann-Pick type C2 (NPC2), which mediated both reduction in cellular cholesterol and antimicrobial activity (8). Other studies have demonstrated the ability of ATRA to restrict infection as well as reduce survival of M. tuberculosis in macrophages by downregulating the expression of tryptophan-aspartate containing coat protein (TACO), a cytoskeletal protein that prevents phagosome-lysosome fusion (9). This ability of ATRA to induce these antimicrobial mechanisms suggests that the generation of ATRA from retinol may be an important factor in host defense against M. tuberculosis infection.

For *in vivo* synthesis of ATRA, retinol is first converted into all-*trans* retinaldehyde (ATRH), a step catalyzed by several enzymes, including short-chain dehydrogenase/reductase family, member 9 (DHRS9), DHRS3, and retinol dehydrogenase 10 (RDH10) (10). ATRH is then converted into ATRA, which can be catalyzed by the aldehyde dehydrogenase 1 (ALHD1) family of enzymes, including ALDH1A1, ALDH1A2, and ALHD1A3 (11). Several of these enzymes are expressed in dendritic cells (DCs), an innate immune cell type which functions as an antigen presentation cell to activate adaptive immune cells and, importantly, is correlated to host immune control of mycobacterial infection (12–19). Although resident DCs exist in normal healthy lung, whether the immune microenvironment in the lung of a TB patient includes DCs or the vitamin A metabolic system is unclear. Therefore, we investigated the potential of innate immune cells to metabolize and activate retinol to elicit vitamin A-driven antimicrobial responses.

## RESULTS

### Activation of innate immune cells by vitamin A metabolites.

**FIG 1:**
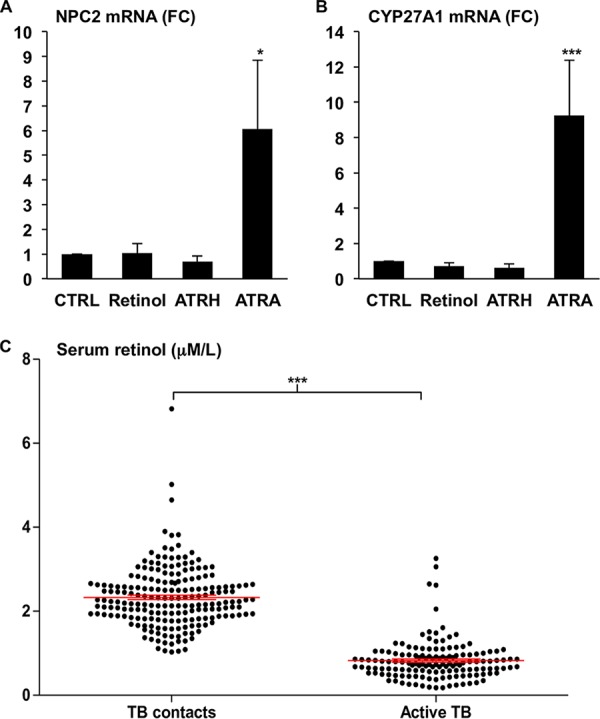
Activation of innate immune cells by vitamin A metabolites. Primary human monocytes were treated with either vehicle alone (CTRL), retinol (10^−8^ M), retinaldehyde (ATRH) (10^−8^ M), or all-*trans* retinoic acid (ATRA) (10^−8^ M) for 18 h and the mRNA expression levels of NPC2 (A) and CYP27A1 (B) were measured via qPCR. Data shown are the average fold change (FC) versus CTRL ± standard error of the mean (SEM) (*n* = 3 to 7). *P* values by one-way ANOVA. *, *P* < 0.05; ***, *P* < 0.001. (C) Black dots represent the serum retinol levels of TB household contact (TB contacts) or active TB patients. The red line indicates the average ± SEM retinol serum levels in each group. *P* value by Student's *t* test. ***, *P* < 0.001.

To determine if retinol or other vitamin A metabolites can directly stimulate monocytes, we stimulated primary human monocytes with equimolar concentrations (10^−8^ M) of retinol, all-*trans* retinaldehyde (ATRH), or all-*trans* retinoic acid (ATRA) for 18 h. Following incubation, total RNA was harvested, and mRNA expression levels of two ATRA response genes, NPC2 and CYP27A1 (8), were measured by real-time semiquantitative PCR (qPCR). Only ATRA stimulation resulted in significant induction of NPC2 mRNA (Fig. 1A), which is a required gene for ATRA-induced antimicrobial activity against M. tuberculosis (8). Similarly, CYP27A1 mRNA expression was significantly induced by ATRA but not by ATRH or retinol (Fig. 1B). However, previous studies have indicated and we confirm here using samples from our completed studies (20, 21) that serum retinol levels were significantly lower in active tuberculosis patients than in healthy household contacts (Fig. 1C). It is then unclear how retinol levels influence tuberculosis pathology; therefore, we hypothesize that local metabolism of retinol into ATRA at the site of infection by immune cells will be crucial to vitamin A-driven host defense.

### GM-DCs express the retinol metabolism pathway.

Since fetal calf serum (FCS) used for cell culture typically contains relatively high levels (∼128 ng/ml) of retinol (22), immune cells cultured in FCS that metabolize retinol to ATRA will subsequently demonstrate an autocrine activation of the retinoic acid receptor (RAR) gene program. Therefore, we sought to interrogate the gene expression profiles of human monocyte-derived macrophages (MDMs) and DCs for a RAR activation signature. Gene signatures of macrophages were determined from our previously published gene microarrays of primary human monocytes stimulated with cytokines that drive macrophage differentiation and polarization (17): interleukin 10 (IL-10; M2a), IL-15 (M1), and IL-4 (M2a). A signature for DCs was determined from a new gene microarray experiment data set of primary human monocytes stimulated with the DC differentiation driving cytokine, granulocyte-macrophage colony-stimulating factor (GM-CSF). Next, Ingenuity Pathway Analysis (IPA) demonstrated that the DC gene signature, but none of the macrophage signatures, had significant enrichment of the canonical pathway “RAR activation” in the GM-CSF-induced gene profile (see Fig. S1 in the supplemental material).

10.1128/mSphere.00327-19.1 FIG S1 Ingenuity Pathway Analysis predicts GM-DCs are cellular source of retinol metabolism. Analysis of the RAR activation gene signature in GM-CSF, IL-10, IL-15, and IL-4 stimulated primary human monocytes. Download FIG S1, PDF file, 0.01 MB. Copyright © 2019 Kim et al. 2019 Kim et al. This content is distributed under the terms of the Creative Commons Attribution 4.0 International license.

**FIG 2:**
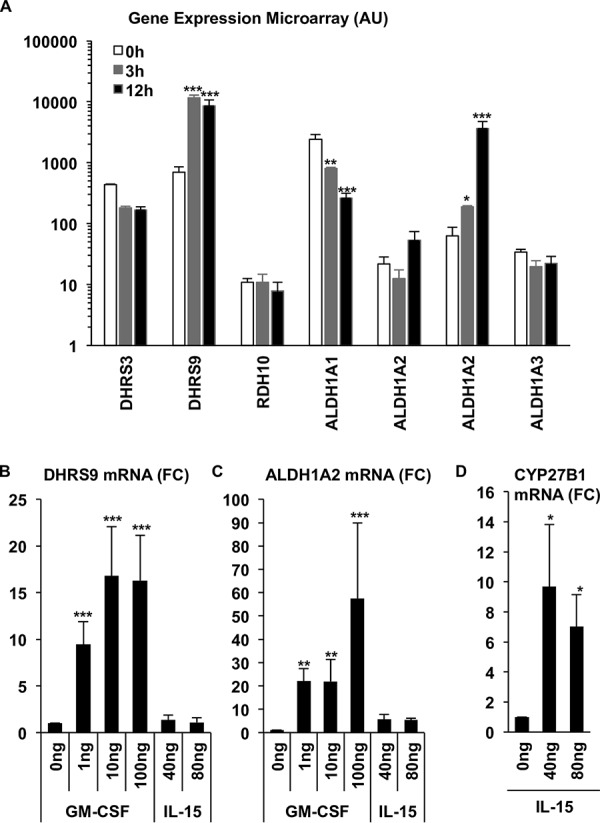
Expression of the retinol metabolic pathway in DCs. (A) Primary human monocytes were stimulated with GM-CSF for 0, 3, and 12 h, and then gene expression was profiled using microarrays. Expression data of vitamin A pathway genes, DHRS3, DHRS9, RDH10, ALDH1A1, ALDH1A2, and ALDH1A3, are displayed as mean expression in arbitrary units (AU) from three independent donors ± SEM. Primary human monocytes stimulated with a titration of GM-CSF or IL-15 for 18 h and mRNA expression of DHRS9 (*n* = 3 to 5) (B) and ALDH1A2 (*n* = 3 to 6) (C) were measured by qPCR. Data shown are the average fold change (FC) versus CTRL ± SEM. (D) Induction of CYP27B1 mRNA expression in monocytes by IL-15 was measured by qPCR. Data shown are the average fold change (FC) versus CTRL ± SEM (*n* = 3). *P* values by one-way ANOVA. *, *P* < 0.05; **, *P* < 0.01; ***, *P* < 0.001.

The retinol metabolism pathway was further elaborated in the DC profile at a single gene level, and the mRNA expression of enzymes that metabolize retinol into ATRH (DHSR3, DHRS9, and RDH10) as well as ATRH into ATRA (ALDH1A1, ALDH1A2, and ALDH1A3) were examined. For the enzymes that metabolize retinol into ATRH, DHSR9 was significantly induced by GM-CSF; however, DHSR3 and RDH10 did not show any significant regulation by GM-CSF (Fig. 2A). As for the enzymes that metabolize ATRH into ATRA, one of two ALDH1A2 probe sets present on the array was significantly upregulated, ALDH1A1 was significantly downregulated, and ALDH1A3 showed no significant regulation by GM-CSF (Fig. 2A). Since both ALDH1A2 probe sets share probes derived from multiple transcripts according to the manufacturer, we designed ALHD1A2-specific primers to confirm the microarray results. Primary human monocytes were isolated from whole blood and stimulated for 24 h with a titration of GM-CSF (0 ng/ml, 1 ng/ml, 10 ng/ml, or 100 ng/ml) compared to a titration of IL-15 (40 ng/ml, 80 ng/ml). Induction of the DC phenotype by GM-CSF was confirmed by the coexpression and increase in cell surface expression of DC-specific markers (23), CD206, CD86, and CD1B measured via flow cytometry (see Fig. S2). The cells were stimulated with IL-15 as a control given that the IPA did not identify an RAR activation signal by IL-15 (Fig. S1), but is known to induce vitamin D metabolism in human monocytes (23). Following the incubation, total RNA was isolated and the expression of DHRS9 and ALDH1A2 was measured by qPCR. GM-CSF at concentrations of 1 ng/ml, 10 ng/ml, and 100 ng/ml induced DHRS9 mRNA levels by 9.43-, 16.8-, and 16.3-fold, respectively, compared to that in an unstimulated control (Fig. 2B). In the same experiment, 1 ng/ml, 10 ng/ml, and 100 ng/ml of GM-CSF induced ALDH1A2 mRNA levels to 22.0-, 21.9-, and 57.4-fold, respectively, compared to that in the unstimulated control (Fig. 2C). Since IL-15 did not induce expression of DHRS9 or ALDH1A2, we assayed CYP27B1 levels to verify that the IL-15 was active, finding that IL-15 induced significant CYP27B1 expression (Fig. 2D) as previously described (23). Concurrent induction of DHRS9 and ALDH1A2 by GM-CSF suggests that the capacity to metabolize retinol into ATRA is part of the GM-CSF-derived DC gene program.

10.1128/mSphere.00327-19.2 FIG S2 GM-CSF induces DC phenotype. Primary human monocytes were differentiated into DCs by stimulation with GM-CSF for 48 h. DC surface markers CD206, CD86, and CD1B were assessed by flow cytometry using monoclonal antibodies (mAB) and their corresponding isotype controls (Iso). Data shown are averages of the mean fluorescence intensity (MFI) ± SEM (*n* = 4). *P* value by Student’s *t* test. **, *P* < 0.01; ***, *P* < 0.001. Download FIG S2, PDF file, 0.06 MB. Copyright © 2019 Kim et al. 2019 Kim et al. This content is distributed under the terms of the Creative Commons Attribution 4.0 International license.

10.1128/mSphere.00327-19.3 FIG S3 Representative isotype control stains for normal and TB lung. Corresponding isotype controls for immunohistochemistry comparing TB lung tissue versus normal lung. (A) Rb IgG isotype control antibody for ALDH1A2; (B) mouse IgG1 isotype control antibody for CD163 and CD1B. Scale bars, 40 μm. Download FIG S3, PDF file, 0.04 MB. Copyright © 2019 Kim et al. 2019 Kim et al. This content is distributed under the terms of the Creative Commons Attribution 4.0 International license.

### Transcriptional profiling of retinol metabolism in TB infected lung.

**FIG 3:**
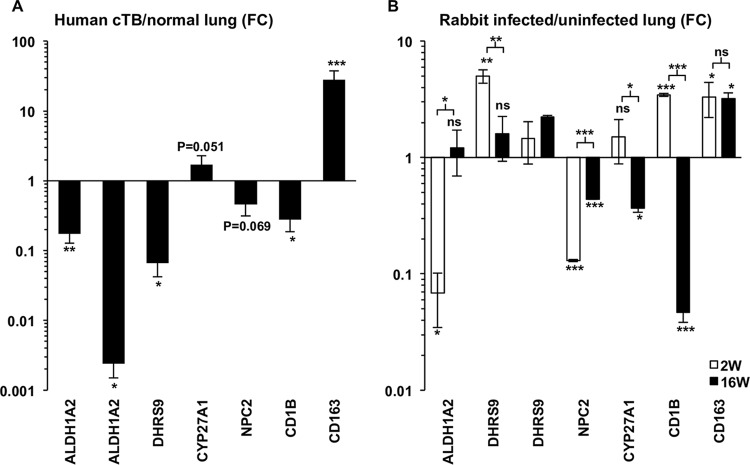
Transcriptional profiling of retinol metabolism in TB infected lung. Expression of vitamin A metabolism (ALDH1A2 and DHRS9) and activation (NPC2 and CYP27A1) genes as well as DC (CD1B) and macrophage (CD163) markers in human caseous TB versus normal lung tissue (A) and rabbit lung 2 and 16 weeks after M. tuberculosis infection (B) as measured by gene microarray. *P* values by one-way ANOVA. *, *P* < 0.05; **, *P* < 0.01; ***, *P* < 0.001.

To establish the *in vivo* relevance of vitamin A metabolism and DCs in TB, we extended our previously published analysis of microarray data, comparing the gene expression profile of human caseous TB lung tissue to that of unaffected lung (24). The expanded analysis indicates that the expression of ALDH1A2 and DHRS9, as well as the DC marker CD1B, was significantly less in TB than in unaffected lung tissue (Fig. 3A). In contrast, the M2 macrophage marker, CD163, was more highly expressed in TB tissue than in unaffected lung (25). These results suggest a lack of a significant vitamin A metabolic gene profile in the chronic and late state of TB disease. To investigate the early stages of infection, we utilized a previously published expression data set comparing rabbit lung after M. tuberculosis infection to uninfected lung tissue (26). This analysis indicates that ALDH1A2 is significantly downregulated after 2 weeks of infection but shows no change after 16 weeks (Fig. 3B). DHRS9 was significantly upregulated at 2 weeks, shown by one of two probes present on the array, but no significant change was detected otherwise (Fig. 3B). NPC2 was significantly downregulated at both 2 and 16 weeks, whereas CYP27A1 was significantly downregulated only at 16 weeks (Fig. 3B). CD1B was significantly upregulated at 2 weeks but significantly downregulated at 16 weeks (Fig. 3B). Lastly, CD163 was significantly upregulated at both 2 and 16 weeks (Fig. 3B). Taken together, with the caveat that the human and rabbit disease pathologies are likely different (27), these data suggest DCs and the vitamin A metabolic pathway are downregulated or absent during the course of tuberculosis infection.

### DCs and retinol metabolism are absent in TB lung.

**FIG 4:**
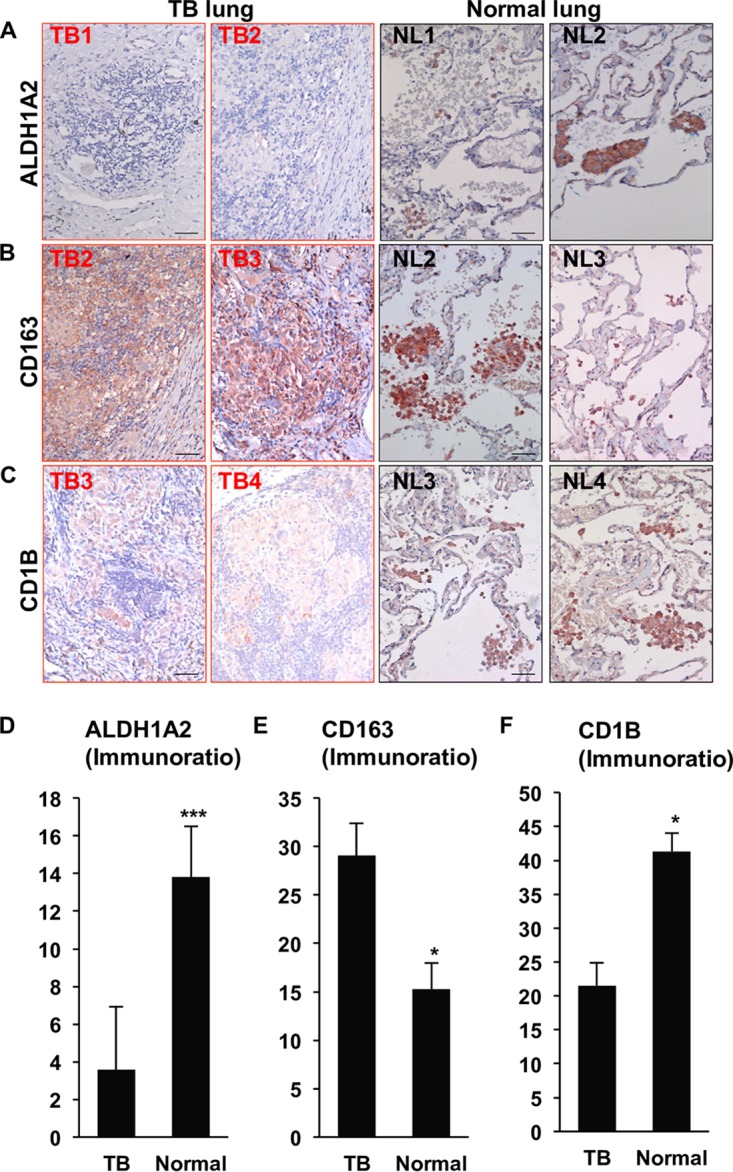
CD1B and ALDH1A2 expression in TB lung. TB patient lung and normal lung tissue protein expression of ALDH1A2 (A), CD163 (B), and CD1B (C) by immunohistochemistry. Data shown are representative images (TB lung [TB], *n* = 8 to 10; normal lung [NL], *n* = 4 to 5). Scale bar, 40 μm. Expression of ALDH1A2 (D), CD163 (E), and CD1B (F) in TB versus normal lung was quantified per nucleated cell using Immunoratio. Data shown are the average percentage of positive cells/nucleus + SEM. *P* value by Student’s *t* test. *, *P* < 0.05; ***, *P* < 0.001.

To confirm the microarray findings, surgically resected lung tissue from five active TB patients were compared to unaffected lung tissue from non-small lung cancer patients obtained by the UCLA Translational Pathology Core Laboratory (TPCL) using immunohistochemistry (IHC). The lung tissues were formalin fixed, paraffin embedded, cut into sections, and mounted onto slides. All TB patient lung sections showed large central caseous necrosis areas surrounded by largely disorganized cellular regions. Within the cellular regions, lung sections from two TB patients (TB1 and TB2) showed little to no ALDH1A2 protein expression, whereas normal lung tissue showed positive cells dispersed throughout the tissue in clusters (Fig. 4A). In terms of cellular composition, both TB and normal lung tissues contained macrophages as detected by CD163 expression (Fig. 4B), but the DC marker, CD1B, was found in the normal lung at higher levels than in TB lung (Fig. 4C). In the normal lung sections, CD163^+^ cells resided predominately in the alveolar space and the border of the interstitium, whereas CD1B^+^ and ALHD1A2^+^ cells were more restricted to the interstitium and endothelium, which is where lung DCs are typically found (28). Corresponding isotype control antibodies for ALDH1A2, CD163, and CD1B show that IHC staining is specific with limited background signal (Fig. S3). Using Immunoratio, we quantified the number of ALDH1A2-, CD163-, and CD1B-positive cells compared to nuclear staining in 4 to 5 sections per normal lung and 8 to 10 sections per TB lung. The decreased mRNA expression levels of ALDH1A2 in TB lung from the microarray data correlated with the decreased protein expression of ALDH1A2 in TB lung relative to normal lung (Fig. 4D). In contrast, we found that TB lung expressed significantly higher CD163 protein levels relative to normal lung (Fig. 4E). Lastly, the decreased CD1B mRNA expression in TB lung also correlated with the decreased protein expression of CD1B in TB lung relative to normal lung (Fig. 4F). Taken together, the decrease in ALDH1A2 and CD1B in the TB lung indicates that genes in the vitamin A metabolic pathway and a DC-specific cell surface marker are significantly lower in TB lung.

### GM-DCs demonstrate metabolism of retinol to ATRA.

**FIG 5:**
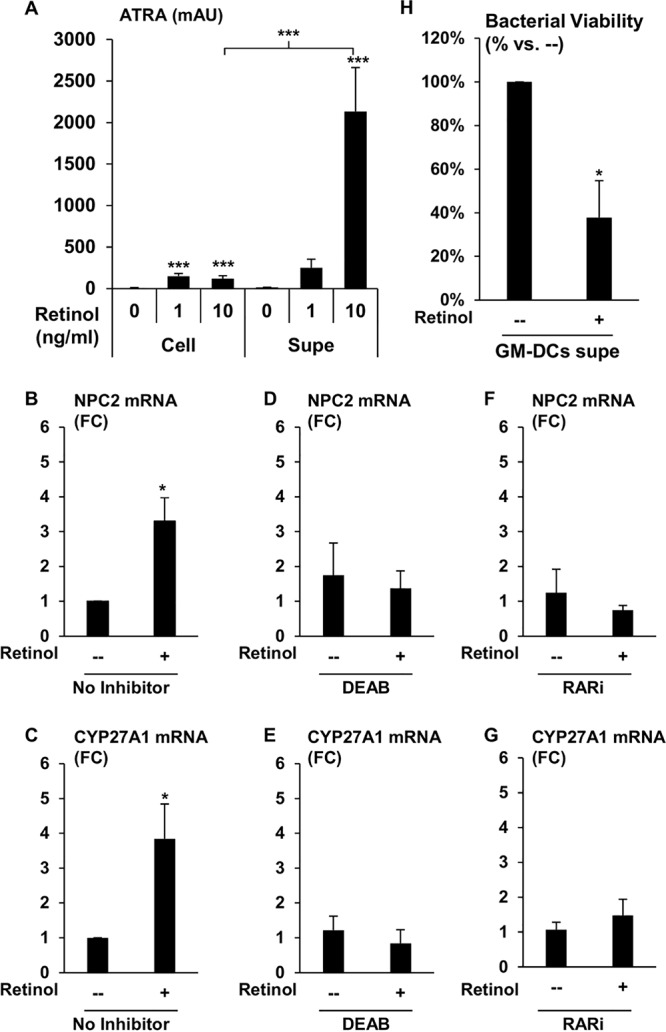
Activation of monocytes and macrophages with DC-produced ATRA. (A) GM-CSF-derived DCs were treated with the indicated amounts of retinol for 6 h under serum-free conditions, and the amounts of ATRA in the cellular (Cell) and supernatant (Supe) fractions were analyzed via HPLC. Data represent the means ± SEMs of the average area of ATRA peak from HPLC plots (*n* = 4). Data shown are the average fold change versus control ± SEM (*n* = 3 to 7). *P* values by one-way ANOVA. *, *P* < 0.05; ***, *P* < 0.001. Expression levels of NPC2 (B) and CYP27A1 (C) were measured by qPCR in primary human monocytes cultured with CM from DCs with or without retinol. From the same experiments, expression levels of NPC2 (D) and CYP27A1 (E) were measured in primary human monocytes cultured with CM from DCs pretreated with DEAB for 20 min prior to addition of retinol. Primary human monocytes were also pretreated with RARi and treated with CM from DC with or without retinol, and mRNA expression levels of NPC2 (F) and CYP27A1 (G) were measured by qPCR. All experimental conditions (panels B to G) were performed in parallel. Data shown are the average fold change versus control ± SEM (*n* = 3 to 7).

For DCs to play a vitamin A-dependent immunomodulatory role in TB, they have to functionally metabolize retinol into ATRA and transactivate infected cells. To establish that the induction of DHRS9 and ALDH1A2 in GM-CSF-derived DCs led to the two-step bioconversion of retinol into ATRA, GM-CSF-derived DCs were cultured under serum-free conditions with or without exogenous retinol for 6 h, and retinol metabolites were measured in the cellular and supernatant fractions via high-performance liquid chromatography (HPLC). The total levels of ATRA were measured from the cellular fraction of DCs incubated with control, 1 ng/ml, or 10 ng/ml of retinol at an average of 9.8, 150.4, and 121.0 mean arbitrary units (mAU), respectively (Fig. 5A). From the same experiments, the ATRA contained in the supernatants from DCs incubated with control, 1 ng/ml, or 10 ng/ml of retinol was quantified at 16.6, 251.2, and 2131.2 mAU, respectively, which at the 10 ng/ml concentration, was significantly (17.6-fold) higher than in the cellular fraction. These data indicate that providing retinol to GM-CSF-derived DCs results in the production of ATRA, which can be found in both the cellular and extracellular fractions, with the majority of ATRA present extracellularly.

### DC-produced ATRA transactivates RAR in monocytes.

To ascertain if the extracellular ATRA produced by DCs can transactivate other immune cells, DCs were cultured for 18 h under serum-free conditions with or without exogenous retinol, and the conditioned medium (CM) supernatants were collected and supplemented with FCS. The supplemented CM was used to culture fresh primary human monocytes for 18 h, and gene expression of NPC2 and CYP27A1 was assessed using qPCR. Incubation of monocytes with the DC CM containing exogenous retinol induced a significant 3.8-fold expression of NPC2 (Fig. 5B) and a 3.3-fold expression of CYP27A1 compared to that in CM with no retinol (Fig. 5C). In the same experiments, we also utilized *N*,*N*-diethylaminobenzaldehyde (DEAB) and BMS 493 (RARi), inhibitors of ALDH1A2 and RAR, respectively, to address the role of vitamin A metabolism and RAR activation in the ability of CM to induce NPC2 and CYP27A1.

Concurrent treatment of the DCs with DEAB during incubation with exogenously added retinol during generation of CM inhibited the ability of the resulting CM to induce NPC2 (Fig. 5D) and CYP27A1 in monocytes (Fig. 5E); however, DEAB had no direct effect on ATRA induction of NPC2 and CYP27A1 (see Fig. S4A). Furthermore, 20-min pretreatment of monocytes with RARi prior to addition of the FCS-supplemented CM also inhibited induction of NPC2 (Fig. 5F) and CYP27A1 (Fig. 5G). In separate experiments, the RARi inhibited ATRA-induced expression of NPC2 and CYP27A1 in primary human monocytes at a 10-fold excess (Fig. S4B). Taken together, these results indicate that DCs are able to produce ATRA, in part through ALDH1A2 activity, from exogenous retinol and stimulate RAR-induced gene expression in neighboring cells.

10.1128/mSphere.00327-19.4 FIG S4 RARi blocked ATRA-induced genes but not DEAB. Primary human monocytes were pretreated with DEAB (A) or RARi (B) at the indicated concentrations and then stimulated with 10^−8^ M ATRA for 18 h. Expression of NPC2 and CYP27A1 was measured by qPCR. Data shown are the average fold change (FC) ± SEM (*n* = 4). *P* values by one-way ANOVA. **, *P* < 0.01; ***, *P* < 0.001. Download FIG S4, PDF file, 0.01 MB. Copyright © 2019 Kim et al. 2019 Kim et al. This content is distributed under the terms of the Creative Commons Attribution 4.0 International license.

### DC-produced ATRA induces antimicrobial activity in M. tuberculosis-infected MDMs.

To determine whether DC-produced ATRA can transactivate innate immune cells to trigger antimicrobial activity, medium was supplemented with or without exogenous retinol (10 ng/ml) and was conditioned by DCs for 18 h. Macrophages were derived from primary human monocytes using M-CSF (MDMs), as we previously described (29), and infected with H37Rv for 18 h. Following the infection, extracellular bacterium was removed through extensive washing, and the infected MDMs were recultured with FCS-supplemented CM. Bacterial viability was assessed after 72 h using our previously described PCR-based method (8). When the infected MDMs were cultured using CM with exogenous retinol, the resulting bacterial viability was significantly lower than for cells cultured with CM with no retinol (Fig. 5H). These data suggest that DCs are capable of triggering a retinol-dependent antimicrobial response in *trans*.

## DISCUSSION

Vitamin A metabolites have been implicated in the pathogenesis of TB in humans. Low levels of the circulating form of vitamin A, retinol, correlate with susceptibility to disease (3, 4, 28), whereas the bioactive form of vitamin A, ATRA, induces antimicrobial activity in M. tuberculosis-infected macrophages (5, 7–9). Given that retinol is biologically inactive, our work sought to explore the mechanism by which retinol can influence the immune response, specifically, to understand how retinol is metabolized into a bioactive form by immune cells. A bioinformatics survey of primary human monocyte gene expression profiles elicited by macrophage- and DC-differentiating cytokines indicated that the DC-differentiating cytokine, GM-CSF, induces expression of genes encoding enzymes in the vitamin A metabolic pathway, DHRS9 and ALDH1A2. Examining human and rabbit TB lung *in situ*, we found that expression of vitamin A metabolism genes and the DC marker CD1B were largely downregulated in lung tissue from TB patients compared to that in controls. GM-CSF-derived DCs, by producing ATRA from retinol and releasing it, can elicit a response in *trans* to neighboring monocytes/macrophages, including induction of NPC2 and an antimicrobial response. Taken together, the present results indicate that DCs, through metabolism of retinol, may play a critical role supplying a bioactive form of vitamin A to neighboring infected macrophages, eliciting antimicrobial responses in TB.

Previous studies have correlated vitamin A deficiency to TB, including populations with a high incidence of TB, such as South Korea (30), India (31), and Indonesia (32). Another multinational study also demonstrated significant association of retinol deficiency with TB in HIV-positive patients (33). Our study examined a multiethnic population in the United Kingdom, which has an overall low incidence of TB and malnutrition (20, 21). The fact that vitamin A deficiency significantly correlated with TB compared to healthy household contacts in our population suggests that retinol plays an important role in TB pathogenesis and may not simply be coincident to malnourishment in impoverished countries with endemic M. tuberculosis or with HIV coinfection. Thus, how retinol is metabolized into the active hormone, ATRA, by the immune system to elicit antimicrobial responses will be an important factor to host defense against M. tuberculosis infection.

The canonical function of DCs is to regulate the adaptive immune response through antigen presentation and cytokine secretion; our results indicate that DCs can also regulate the immune response through metabolic activity. Here, we provide evidence that metabolism of vitamin A by DC leads to the release of ATRA, which can subsequently transactivate RAR in neighboring monocytes/macrophages. Transcellular metabolism was previously described for the production of eicosanoids from arachidonic acid, where prostaglandins are metabolized by one cell and then the biosynthetic intermediates are delivered to monocytes and macrophages to be further metabolized (34, 35). In contrast, our results provide evidence that the metabolism of retinol into ATRA by DCs led to transactivation of macrophages, resulting in antimicrobial activity against M. tuberculosis. There is increasing evidence that nuclear hormones such as vitamin A and vitamin D play an integral role in innate immune response, and while both require a metabolic step converting the inactive substrate into the active hormone, it is unclear if vitamin D metabolism results in transcellular activation (36).

The role of DCs in TB pathogenesis is not well defined due to the lack of an accessible experimental animal system that accurately models the human disease as well as to the difficulty in acquiring lung tissue samples from active and latent TB patients. Many of the immune paradigms described for the host immune response to mycobacterial infections have, therefore, been established by studying other human mycobacterial diseases, especially leprosy, which is caused by dermal infection with Mycobacterium leprae. CD1B^+^ DCs were found in the granulomas derived from patients with the self-limiting form of leprosy (tuberculoid); in contrast, DCs were absent in the lesions derived from patients with the disseminated form of leprosy (lepromatous) (37). Examination of resected lung tissue from patients with active TB confirmed similar results, since both gene and protein expression of the DC specific marker, CD1B, as well as ALDH1A2 are significantly diminished in TB lung relative to that in normal lung, accompanied by an increase in CD163 expression (25). Although CD163 is predominantly expressed by macrophages, a subset of tolerogenic IL-10-secreting DCs expressing cell surface CD163 have been described (38). The presence of IL-10-secreting tolerogenic DCs would correlate with the permissive immune microenvironment of both lepromatous leprosy and tuberculosis and warrants further investigation of their role in disease pathogenesis (39, 40).

Since patient-derived TB lung samples represent a late stage of disease, we utilized data from experimental animal studies to understand the regulation of DCs and vitamin A metabolism at the onset of infection in rabbits. Based on our gene expression analysis of rabbit lung, while there was an increase in CD1B expression at the onset of M. tuberculosis infection, the levels dropped as the infection progressed. Other studies have shown a similar pattern in the mouse lung during M. tuberculosis infection, where DCs drain to the nearest lymph node and have impaired function (41). However, the mechanism leading to the decrease in CD1B and ALDH1A2 in human TB lung is unclear given M. tuberculosis infection has been described to result in DC cell death (42), transmigration away from the lung (43), and defective DC differentiation (44). Our previous study indicates that ATRA stimulation does not induce CD1B expression (45), but we cannot rule out that ATRA might result in the downregulation of CD1B on the cell surface of DCs. However, given that TB patients are likely to be systemically retinol deficient and possibility ALDH1A2 deficient at the site of infection, it is unlikely there will be abundant ATRA present. Since the presence of DCs and likely their ability to function properly correlates with favorable outcome to disease, factors that regulate DCs during infection in the complex microenvironment of granulomas will warrant further investigation. Although at this time, we cannot differentiate if DCs in the TB lung have downregulated expression of CD1B and ALDH1A2 but are still present nor rule out the presence of DCs with low CD1B and ALDH1A2 expression.

We demonstrate here that generation of ATRA by DCs can transactivate infected macrophages to elicit antimicrobial activity. However, due to the pleiotropic effects of ATRA on the immune system, the effects of ATRA transactivation are not limited to induction of NPC2, and include other processes critical to host defense against M. tuberculosis infection, such as (i) autophagy (46), (ii) driving monocyte differentiation into CD209^+^ macrophages with antimicrobial properties (45), or (iii) macrophages with tissue-preservation/wound-healing functions (47). ATRA can also regulate the adaptive immune response through inducing differentiation of Foxp3^+^ regulatory T cells as well as preserving Th1 inflammatory responses (48). Since sustained inflammation in the lung can lead to severe lung tissue damage or mortality, regulation of chronic inflammation by FoxP3^+^ regulatory T cells can be important in disease outcome and reduction of mortality (49). In combination, CD209^+^ macrophages and the Th1 inflammatory response are critical to the clearance of mycobacterium from the host (29, 50, 51), whereas tissue-like macrophages are critical to sustaining granulomas, thus preventing the spread of disease within the host *in vivo* (52). Studies have also indicated that ATRA regulates the composition of the extracellular matrix through induction of a gene expression profile that drives tissue preservation and wound healing. Active TB lungs show an increase of MMP-1, MMP-8, and MMP-9 protein expression and a decrease in TIMP-1 protein expression, which results in pulmonary cavitation (53–55). In contrast, stimulation of monocytes with ATRA decreases mRNA expression of MMP-1/MMP-9 and increases the expression of TIMP-1, leading to tissue preservation and wound healing (47). The diverse roles of ATRA-mediated immunological pathways suggest that regulating vitamin A metabolism at the site of infection can play a critical role in balancing the antimicrobial response and mitigate inflammation-triggered tissue damage in TB.

Clinical studies have shown that patients with active TB have lower circulating retinol levels than healthy individuals (2–4); however, retinol supplementation has demonstrated mixed results (56–60). Yet, Aibana et al. demonstrated that household contacts who are vitamin A deficient have a 10-fold increased risk in acquiring tuberculosis disease (2), and active TB patients treated with chemotherapy exhibit increased circulating retinol levels (4). These studies indicate that the vitamin A system plays an important role in mitigating TB; however, the mechanism by which retinol influences the immune response is not a direct effect. Our results here demonstrate that for retinol to trigger an antimicrobial response, it must first be metabolized into ATRA; therefore, since TB lung has lower expression of ALDH1A2, systemic restoration of retinol levels will have minimal effects at the site of infection with a lack of local metabolism-dependent transactivation. Therefore, novel therapeutics that enhance retinol metabolism in the lungs of active TB patients could potentially be used in conjunction with retinol supplementation as an immunotherapy targeting the innate immune response to infection. Alternatively, given that normal lung expresses the vitamin A metabolic machinery, supplementation of retinol-deficient individuals prior to M. tuberculosis exposure provides a strategy that might prevent the spread of disease.

## MATERIALS AND METHODS

### Statistics.

A two-tailed Student's *t* test was used to compare two different experimental conditions. Experiments with three or more measurements were analyzed using one-way analysis of variance (ANOVA) or Kruskal-Wallis one-way ANOVA on ranks as appropriate with the Student-Newman-Keuls method for pairwise analyses using GraphPad Prism 7 software. Error bars in figures represent the standard errors of the means between individual donor values. The *P* values are either precisely indicated in the text or noted in the figures using the following convention: *, *P* < 0.05; **, *P* < 0.01; ***, *P* < 0.001.

### Reagents.

Retinol, ATRA, and ATRH were purchased (Sigma-Aldrich), dissolved in dimethyl sulfoxide (DMSO) and stored at −80°C in small aliquots protected from light. Unless stated, the retinol, ATRH, and ATRA were utilized at 10^−8^ M. Recombinant IL-15 (R&D systems) and GM-CSF (R&D systems) were cultured with monocytes in RPMI 1640 (Gibco) and 10% fetal calf serum (FCS) (Omega Scientific). Monocytes were differentiated into macrophages with macrophage colony-stimulating factor (M-CSF) (R&D systems) as described as previously (8). Immunohistochemistry was performed with the following antibodies: purified-CD1B (MT101; BD biosciences), purified-ALDH1A2 (ab75674; Abcam), and purified-CD163 (EDHu-1; Bio-Rad).

### Retinol measurements of clinical samples.

Adult TB contacts (*n* = 202) and adult patients with smear-positive pulmonary TB (*n* = 145) were recruited from TB clinics in London, UK, as previously described (20, 21). Serum concentrations of retinol were determined by high-performance liquid chromatography (HPLC) in the clinical biochemistry departments of Northwick Park Hospital (TB contacts) and the Royal London Hospital (TB patients). The studies were approved by the research ethics committees of North East London, Harrow, and East London and The City Research Ethics Committee (REC references P/02/146, EC 2759, and 06/Q0605/83), and written informed consent to participate was obtained from all participants.

### Flow cytometry.

The following antibodies (BD Biosciences) were used for flow cytometry: CD1B-fluorescein isothiocyanate (FITC; MT101), CD86-allophycocyanin (APC; FUN-1), and CD206-phycoerythrin (PE; 19.2). DCs were harvested and subsequently stained as previously described (15).

### Quantitative real-time PCR.

Gene expression of CYP27B1, CYP27A1, NPC2, DHRS9 and ALDH1A2 were analyzed by real-time semiquantitative PCR (qPCR) as described previously (8). The primers were as follows: ALDH1A2 Forward, 5′-TTG GTT CAG TGT GGA GAA GG-3′; ALDH1A2 Reverse, 5′-AAA GCT TGC AGG AAT GGT TTG-3′; DHRS9 Forward, 5′-CTT GCA ATC GTT GGA GGG GGC T-3′; DHRS9 Reverse, 5′-AGA CAG CTG CTC CCA AAT GGC G-3′. Genes were normalized to 36B4 levels and the threshold cycle (ΔΔ*C_T_*) method was used to calculate fold change; 1 mg of RNA was used.

### Bioconversion of retinol to all-*trans* retinoic acid.

DCs were differentiated as described above and treated with or without retinol for 6 h under serum-free conditions. The cultures were harvested, and the supernatants and cellular fractions were separated by centrifugation (300 × *g*). Retinoids were extracted in hexane, and samples were analyzed by high-performance liquid chromatography as previously described (61). ATRA levels are expressed as mean arbitrary units (mAU).

### Conditioned medium.

DCs were generated from primary human monocytes treated with GM-CSF for 2 days. The DCs were harvested, washed, enumerated, and a portion was pretreated with DEAB (STEMCELL Technologies) for 20 min; all the DCs were treated with or without retinol for 18 h at 37°C and 5% CO_2_. The conditioned medium was harvested, filtered with a 0.2-μm filter, aliquoted, and frozen in a −80°C freezer. Fresh monocytes were then isolated by plastic adherence, and a portion were pretreated with BMS493, a pan-RAR inhibitor (RARi) (Tocris), for 20 min at 37°C and 5% CO_2_; then, 900 μl of conditioned medium and 100 μl of fresh FCS were added to the culture. The cells were incubated for 18 h and the gene expression of NPC2 was measured as explained above.

### M. tuberculosis and antimicrobial assay.

H37rv was cultured, harvested, and enumerated as previously described (8). Macrophages were isolated as explained above and infected with H37rv for 24 h. Extracellular bacteria were vigorously washed out of the tissue culture well, and the cells were treated with 90% of conditioned medium and 10% of fresh FCS and incubated for 3 days. The monolayers were harvested, and bacterial viability was calculated with a PCR-based method as previously described (8), which compares 16S RNA levels to a genomic DNA (IS*6110*) levels as an indicator of bacterial viability. Following the incubation, the cells are harvested and divided. Half of the cells were lysed by boiling at 100°C for 5 min and then snap-frozen at −80°C. Total RNA was isolated from the remaining half using TRIzol (Life Technologies) according to the manufacturer’s recommended protocol, followed by RNA cleanup and on-column DNase digestion using an RNeasy Miniprep kit (Qiagen, Valencia, CA). cDNA was synthesized from the total RNA using the iScript cDNA Synthesis kit (Bio-Rad, Hercules, CA) according to the manufacturer’s recommended protocol. The bacterial 16S rRNA and genomic element DNA levels were then assessed from the cDNA and cellular lysate, respectively, by real-time PCR using iQ SYBR green (Bio-Rad). Comparison of the bacterial DNA to the mammalian genomic 36B4 levels was used to monitor infectivity between all the conditions in the assay as well as PCR quality. The 16S and DNA values were calculated using the ΔΔ*C_T_* analysis, with the mammalian bacterial DNA value serving as the housekeeping gene.

### Caseous versus human lung microarray data.

For the caseous tuberculosis granuloma, data files were obtained from the Gene Expression Omnibus database (https://www.ncbi.nlm.nih.gov/geo/, accession numbers GSE20050, GSE23073, GSE13762, and GSE28995) (62). Gene expression levels were normalized to G3PDH. Since there are multiple G3PDH probes represented on the microarray, the NPC2 and IL-6 probe values were normalized to every G3PDH probe and averaged.

### Microarray analysis of macrophage and DC profiles.

Expression profiles of IL-10-, IL-15-, and IL-4-derived macrophages were analyzed from previous data deposited in the Gene Expression Omnibus database (https://www.ncbi.nlm.nih.gov/geo/) in series entity GSE59184 (17). Briefly, adherent peripheral blood mononuclear cells (PBMCs) from four healthy individuals were stimulated with the various cytokines, and CD14^+^ cells were harvested at 0 h, 6 h, or 24 h after stimulation. RNA expression was analyzed by Affymetrix Human U133 Plus 2.0 array. DC expression profiles were derived from CD14^+^ purified monocytes from three healthy individuals and then stimulated with recombinant GM-CSF (100 U/ml) for 0, 3 h, or 12 h. Total RNA was isolated and then processed by the University of California Los Angeles Clinical Microarray Core Facility using Affymetrix Human U133 Plus 2.0 array and normalized as previously described (8, 36). Expression gene signatures of DCs and macrophages were determined by analyzing genes with ≥1.5-fold change, a *P* value of ≤0.05, and experimental minus baseline intensity of ≤100 across all time points versus time zero. Gene signatures were then analyzed by Ingenuity Pathways Analysis (Qiagen) using the canonical pathways comparison function for nuclear receptor activation. Microarray data from rabbit lung was downloaded from GSE33094 (26). Briefly, New Zealand White rabbits were infected via aerosol with M. tuberculosis HN878, and total RNA isolated from rabbit lungs at indicated times. Agilent-020908 Oryctolagus cuniculus two-color Oligo Microarray was utilized using one channel for infected and the other color channel for uninfected per sample. Gene expression was represented as the fold-change between M. tuberculosis HN878-infected and uninfected animals at various times postinfection.

### Immunohistochemistry.

The tissue sections were deparaffinized using xylene washes for 5 min each followed by three 10-min 100% ethanol washes. After the washes, the slides were placed in a pressure cooker containing 15 ml of antigen unmasking solution (vector) in 1,600 ml of distilled water at a boil. The slides were incubated in the pressure cooker at full pressure for 5 min and then placed in phosphate-buffered saline (PBS) to prevent the tissue from drying. Then, the slides were transferred into 3% H_2_O_2_ for 10 min followed by three washes with distilled water. For ALDH1A2, the tissue sections were blocked with PBS-5% rabbit serum, and for CD1B, PBS-5% goat serum, for 30 min at room temperature; then, the sections were incubated with the corresponding primary antibody at the manufacturer’s recommended concentration at 4°C overnight. A biotinylated secondary antibody corresponding to the species type of the primary antibody was added to the sections following washes for 1 h at room temperature. The slides were developed with ABC reagent (Vector) for 30 min at room temperature and then treated with peroxidase solution for 30 min at room temperature. The tissue sections were counterstained with hematoxylin.

### Study approval.

This study was conducted according to the principles expressed in the Declaration of Helsinki and was approved by the Institutional Review Board of the University of California at Los Angeles. Whole blood from healthy donors was acquired from two sources: (i) through the UCLA CFAR Virology Core and (ii) UCLA IRB number 92-10-591-31 with informed consent. Peripheral blood mononuclear cells (PBMCs) were isolated from the peripheral blood of healthy donors using a Ficoll-Paque (GE Health Care) density gradient, and monocytes were purified by plastic adherence as previously described (8, 15, 23). Monocyte-derived macrophages (MDMs) and granulocyte-macrophage colony-stimulating factor (GM-CSF)-derived DCs were produced as previously described (15, 23).
