# Supplementary figures and images for: Vitamin A Metabolism by Dendritic Cells Triggers an Antimicrobial Response against Mycobacterium tuberculosis
Source: mSphere. 2019 Jun 5;4(3):e00327-19. doi: 10.1128/mSphere.00327-19 (PMC6553556; doi:10.1128/mSphere.00327-19)

Figure S1

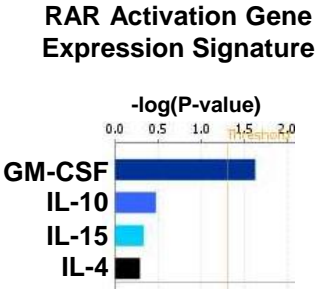

Supplement: FIG S1 [file mSphere.00327-19-sf001.pdf]

Fig. S2

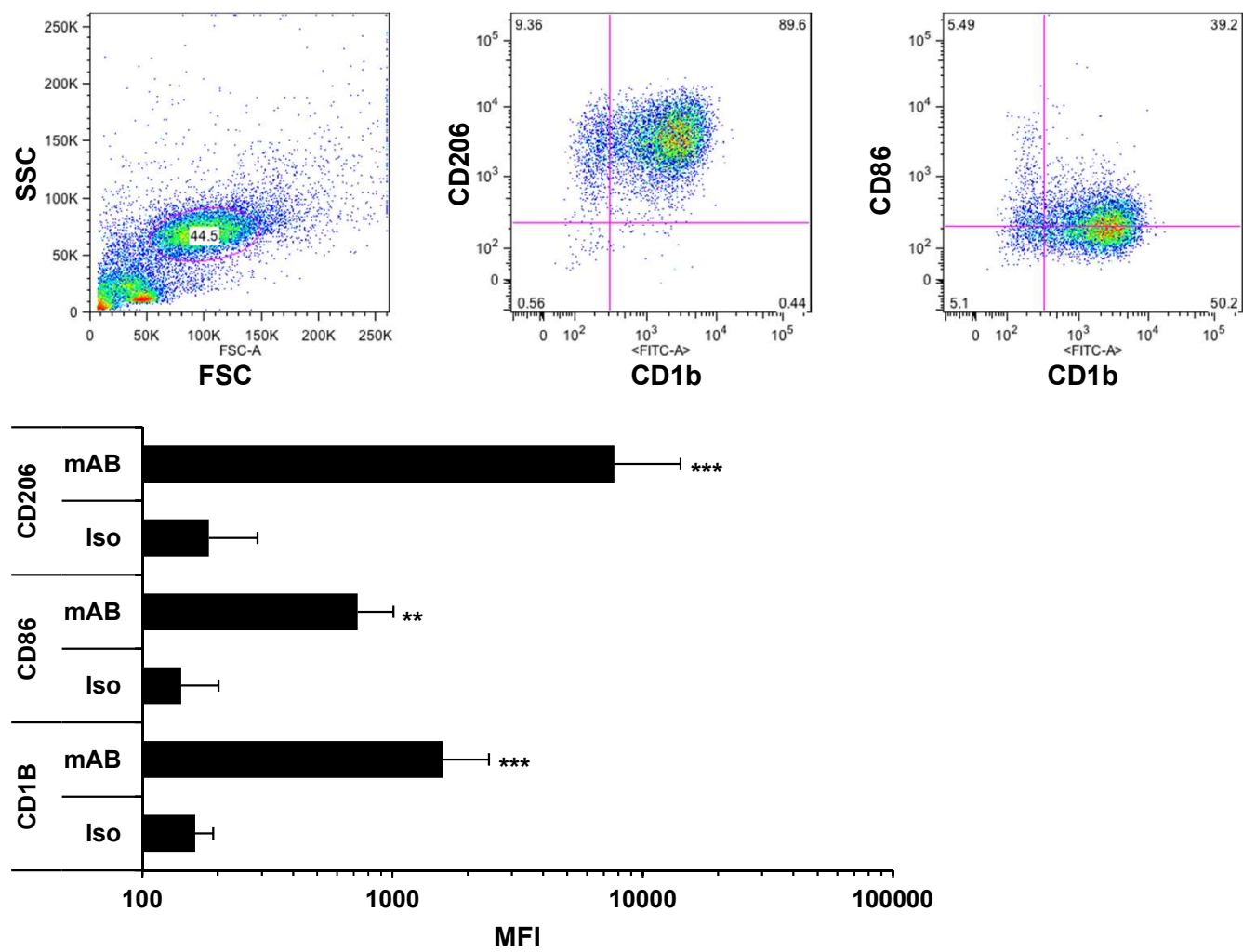

Supplement: FIG S2 [file mSphere.00327-19-sf002.pdf]

Fig. S3

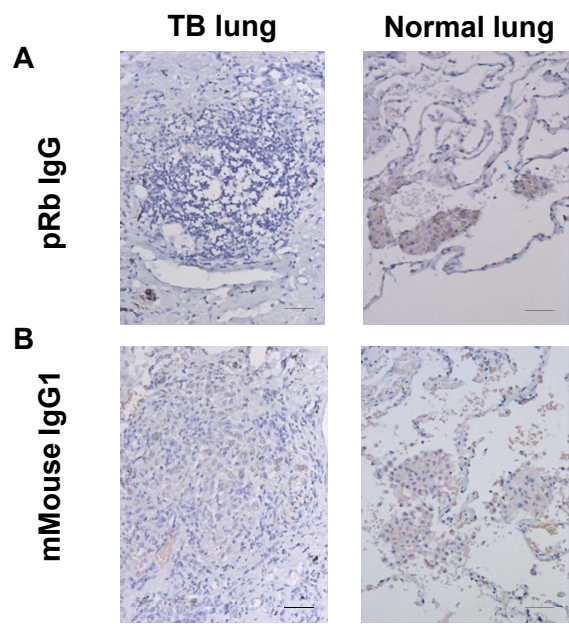

Supplement: FIG S3 [file mSphere.00327-19-sf003.pdf]

Figure S4

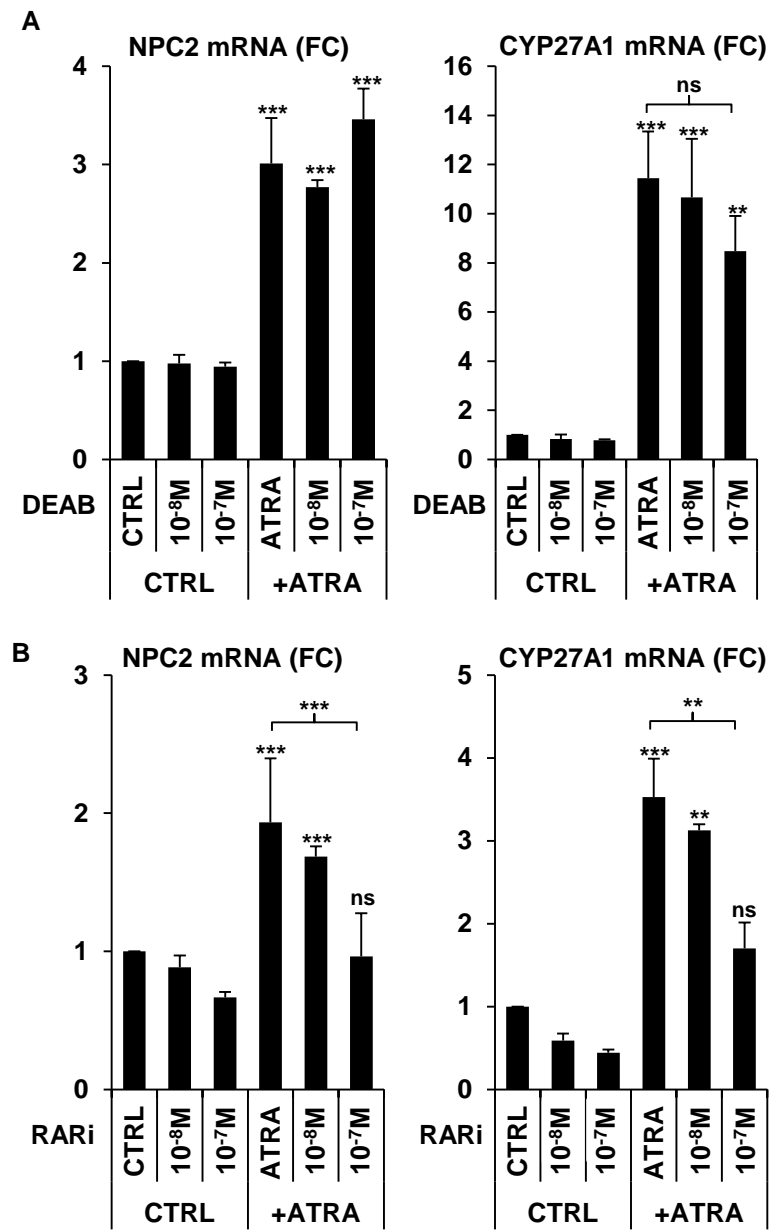

Supplement: FIG S4 [file mSphere.00327-19-sf004.pdf]
